# Construction of an Immune Cell Infiltration Score to Evaluate the Prognosis and Therapeutic Efficacy of Ovarian Cancer Patients

**DOI:** 10.3389/fimmu.2021.751594

**Published:** 2021-10-20

**Authors:** Jinhui Liu, Yichun Wang, Shuning Yuan, Junting Wei, Jianling Bai

**Affiliations:** ^1^ Department of Gynecology, The First Affiliated Hospital of Nanjing Medical University, Nanjing, China; ^2^ Department of Urology, The First Affiliated Hospital of Nanjing Medical University, Nanjing, China; ^3^ The Second Clinical School of Nanjing Medical University, Nanjing, China; ^4^ Department of Biostatistics, School of Public Heath, Nanjing Medical University, Nanjing, China

**Keywords:** ovarian cancer, ICI score, immunotherapy, chemotherapy, tumor immune microenvironment

## Abstract

**Background:**

Ovarian cancer (OC) is an immunogenetic disease that contains tumor-infiltrating lymphocytes (TILs), and immunotherapy has become a novel treatment for OC. With the development of next-generation sequencing (NGS), profiles of gene expression and comprehensive landscape of immune cells can be applied to predict clinical outcome and response to immunotherapy.

**Methods:**

We obtained data from The Cancer Genome Atlas (TCGA) and Gene Expression Omnibus (GEO) databases and applied two computational algorithms (CIBERSORT and ESTIMATE) for consensus clustering of immune cells. Patients were divided into two subtypes using immune cell infiltration (ICI) levels. Then, differentially expressed genes (DEGs) associated with immune cell infiltration (ICI) level were identified. We also constructed ICI score after principle-component analysis (PCA) for dimension reduction.

**Results:**

Patients in ICI cluster B had better survival than those in ICI cluster A. After construction of ICI score, we found that high ICI score had better clinical OS and significantly higher tumor mutation burden (TMB). According to the expression of immune checkpoints, the results showed that patients in high ICI group showed high expression of CTLA4, PD1, PD-L1, and PD-L2, which implies that they might benefit from immunotherapy. Besides, patients in high ICI group showed higher sensitivity to two first-line chemotherapy drugs (Paclitaxel and Cisplatin).

**Conclusion:**

ICI score is an effective prognosis-related biomarker for OC and can provide valuable information on the potential response to immunotherapy.

## Introduction

Ovarian cancer (OC), a rare disease among women, accounts for approximately 300,000 new cases and 180,000 deaths worldwide every year (1). The epithelial ovarian carcinoma presents as the most common pathological type of OC (2). It is widely acknowledged that only 20% of the patients have poor prognosis due to the high proportion of women diagnosed with stage III–IV disease and high recurrence rate (3). Nowadays, immunotherapy has become a novel treatment to benefit patients with better outcome (4), with fast-track approvals in various malignant tumors except OC (5). The usage of immunotherapy will bring new sight into the treatment of OC and provide hope for patients with poor prognosis.

There has been mounting evidence that immunological destruction of tumors can be targeted at several points to achieve clinical remission (6, 7). Over the past decade, researchers have used blocking antibodies to programmed cell death 1 (PD-1) and cytotoxic T-lymphocyte-associated protein-4 (CTLA-4) to reactivate the immune system and eliminate cancer cells (8–10).

It has been recognized that OC belongs to immunogenetic diseases and contains tumor-infiltrating lymphocytes (TILs) (11, 12), which is a potential therapeutic biomarker for immune-checkpoint blockade therapy (4, 13). However, only modest treatment performance has been observed for single agent immune-checkpoints inhibitors (ICIs) in OC (14, 15), although multiple studies showed that patients with rich T-cell tumors exhibited good prognosis (11). Thus, it is important to establish a scoring system to filter the potential patients who may benefit from immunotherapy (16).

Immune-suppressive networks have become a major challenge in applying immunotherapy in OC patients (12, 17). Several studies have revealed potential immune resistance mechanisms in OC, and next-generation sequencing (NGS) technology has been applied in several malignancies including OC (18, 19). In our study, with CIBERSORT and ESTIMATE algorithms (20, 21), we analyzed profiles of gene expression and obtained a comprehensive landscape of immune cells. Based on infiltration of immune cells, two distinct subtypes were divided using the OC cohorts. We then established and validated the ICI score in OC patients to predict clinical outcome and response to immunotherapy.

## Materials and Methods

**Figure 1:**
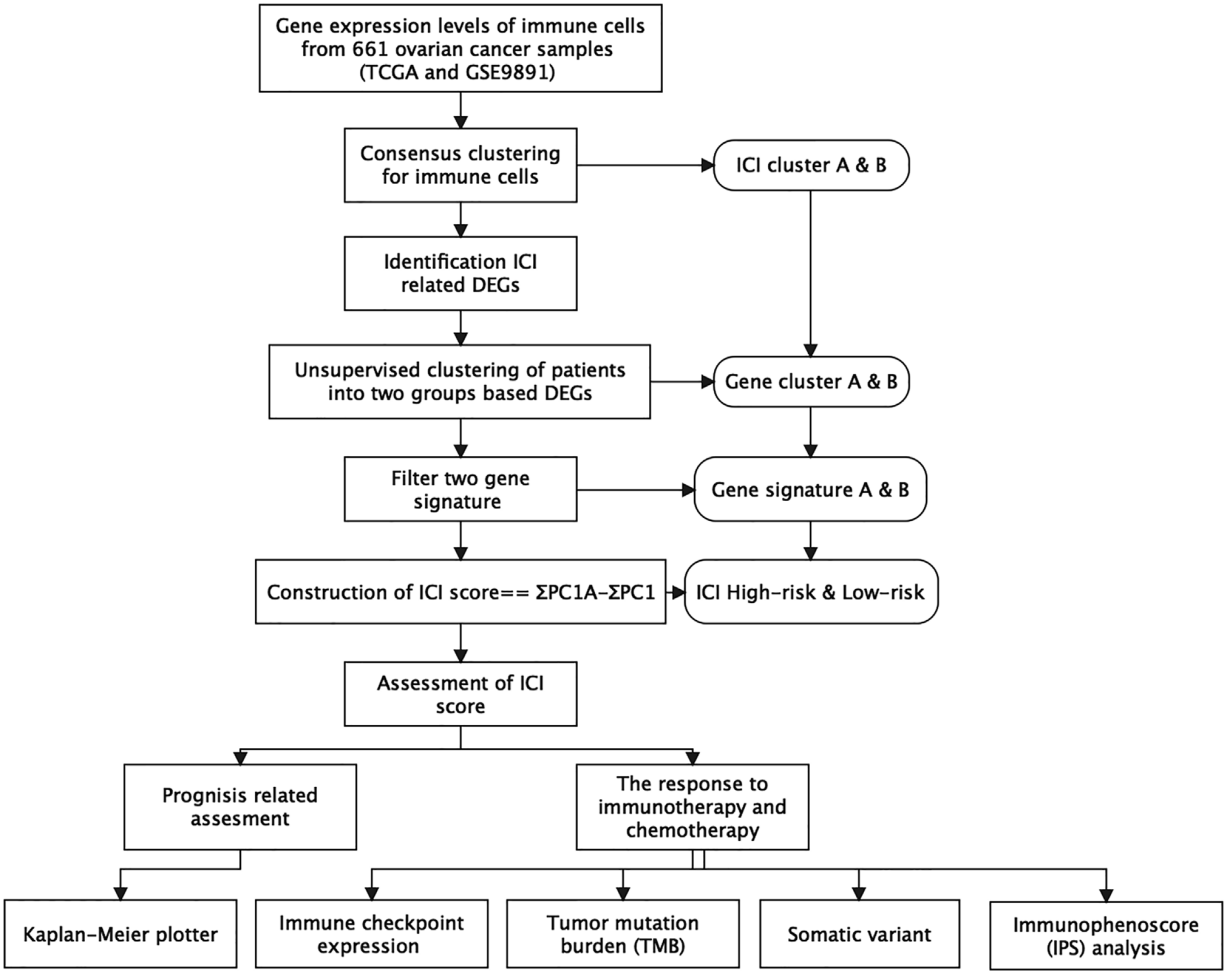
The brief workflow of this research.

The brief workflow of this research is exhibited in **Figure 1**. We obtained the gene expression levels of immune cells from The Cancer Genome Atlas (TCGA) and Gene Expression Omnibus (GEO) databases, followed by consensus clustering for these immune cells. Based on the differentially expressed genes (DEGs) identified, we conducted unsupervised clustering to separate patients into two genomic clusters (gene clusters A and B). Among them, genes were defined as ICI gene signature A for positive association with gene cluster, while other genes that showed negative association with gene cluster were defined as ICI gene signature B. Then, principle-component analysis (PCA) was conducted to calculate the score of ICI gene signature with ICI ΣPC1_A_ – ΣPC1_B_. The detailed description of the full method is listed in the following sections.

### Ovarian Cancer Cohorts and Processing

A total of 379 ovarian cancer samples were obtained from the TCGA (http://cancergenome.nih.gov), including RNA-sequencing transcriptomic, genetic mutations (VarScan) and clinical data. Microarray data (GSE9891) were downloaded from GEO (https://www.ncbi.nlm.nih.gov/geo). Patients without full survival information were excluded from our cohort. The fragments per kilobase of transcript per million mapped reads (FPKM) values of TCGA-OV dataset were transformed to transcripts per kilo base million (TPM) values as previously reported (22). ComBat algorithm was utilized to reduce the batch effects caused by non-biotech bias between different datasets (23).

### Consensus Clustering for Immune Cells

Gene expression levels of immune cells in ovarian cancer were quantified using *CIBERSORT* R package (24), and *ESTIMATE* algorithm was used to evaluate tumor purity, stromal, and immune score. Then, the hierarchical agglomerative clustering of OC was performed in accordance with ICI pattern (25). We conducted the ConsensuClusterPlus package to execute the steps above and repeated 1,000 times to provide stabilized classification results (25).

### DEGs Associated With ICI Phenotype

We divided patients into two ICI clusters based on the infiltration level of immune cells to distinguish differential genes that are related to ICI pattern. Using limma R, significant cutoff criteria were established as adjusted *p <*0.05 (26). Then, ClusterProfiler R package was employed to perform functional annotation for every gene with cutoff value of false discovery rate (FDR) <0.05 (27).

### Dimension Reduction and Construction of ICI Score

We conducted unsupervised clustering in our study to separate patients into genomic clusters (gene clusters A and B) according to DEG values. ICI gene signatures A and B were constructed and included genes that were positively or negatively associated with the clusters, respectively. Then, we performed Boruta algorithm to reduce dimension in the ICI gene signatures A and B (28). For variables of ICI landscape in OC patients, PCA was employed to extract first principal component as the signature score. Construction of ICI score of every patient was built using a method similar to the gene expression grade index (29) with ICI ΣPC1_A_ – ΣPC1_B_. We classified patients based on ICI scores using the surv-cutpoint function from the survival package.

### Construction and Validation of a Predictive Nomogram

To fully expand the predictive power of ICI score, we constructed a nomogram based on the clinical characteristics of OC, including age, grade, and stage. The calibration plot was used to validate the nomogram.

### Data Collection of Somatic Variants

Considering the potential of TMB in predicting response of immunotherapy, we performed a stratified survival analysis that explored the relationship between TMB score and ICI score. Relevant data of somatic variants in ovarian cancer patients were downloaded from TCGA data portal (https://www.cancer.gov/tcga/). Mutation data were analyzed using the maftools packages in R (30). We chose top 20 genes that had the highest possibility to be mutated.

### Immunophenoscore Analysis and Chemotherapy

Immunophenoscore (IPS), consisting of MHC molecular (MHC), effector cells (ECs), immune checkpoints (CPs), and immunosuppressive cells (SCs), can be generated in an unbiased manner using machine learning methods based on four major gene categories that determine immunogenicity. Immunophenotype scores with a scale of 0–10 were calculated using expression values of representative genes or immune manifestation of gene sets. We obtained the IPS of OC patients from the Cancer Immunome Atlas (TCIA) (https://tcia.at/home) (31). The half-maximal inhibitory concentration (IC50) of selected drugs was estimated from a public database called Genomics of Drug Sensitivity in Cancer (GDSC; https://www.cancerrxgene.org) (32).

### Single-Sample Gene-Set Enrichment Analysis

To quantify the immune activity of different groups, single-sample gene-set enrichment analysis (ssGSEA) was used to explore gene signatures (33).

### Verification of the ICI Score

We included an independent anti-PD-L1 immunotherapy cohort (IMvigor210) to verify whether ICI score can predict the response to immunotherapy. We used IMvigor219CoreBiologies R package to obtain the expression and clinical data (34).

### Statistical Analysis

All statistical analyses were conducted using R version (4.1.0). Wilcoxon test was applied to compare two groups, and Kruskal–Wallis test was used to compare more than two groups. We utilized the Kaplan–Meier plotter to generate survival curves for subgroups in each dataset, and their differences were evaluated using the log-rank test. The surv-cutpoint function from the survival package was applied to stratify samples into high and low ICI subgroups. The chi-square test analyzed the Spearman correlation between the ICI score subgroups and somatic mutation frequency. Two-tailed *p* < 0.05 was considered as statistically significant.

## Results

### Identification of Two Immune Cell Infiltration-Related Clusters

**Figure 2:**
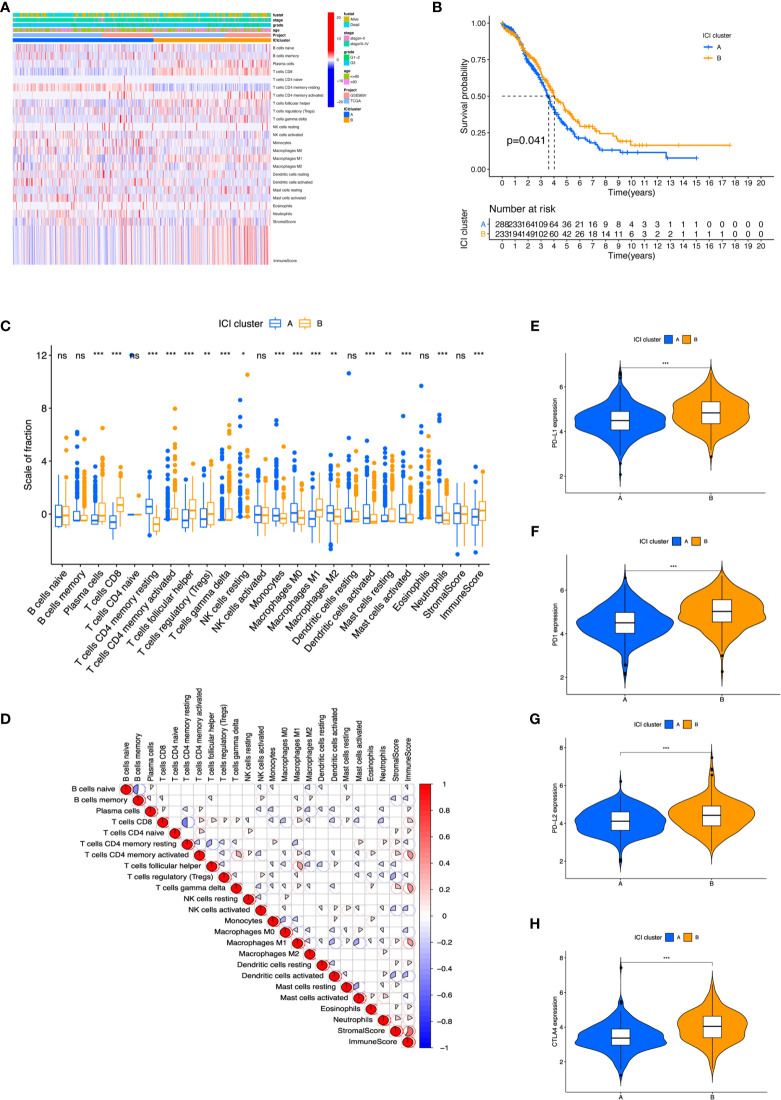
The landscape of Immune cells infiltration in the TME of OC. **(A)** Heatmap representing unsupervised clustering of infiltrating immune cells in two OC cohorts. **(B)** Kaplan–Meier curves showed OS of all patients between two ICI clusters. **(C)** The fraction of tumor-infiltrating immune cells and immune and stromal scores in the two ICI clusters. **(D)** Cellular fraction of the tumor-infiltrating immune cell types in ICI clusters. **(E–H)** Difference in PD-L1 **(E)**, PD1 **(F)**, PD-L2 **(G)**, and CTLA4 **(H)** expression between ICI clusters. *p < 0.05; **p < 0.01; ***p < 0.001; ns, not significant.

CIBERSORT and ESTIMATE algorithms were first conducted to quantify enrichment levels of immune cells in ovarian cancer tissues with a total of 661 samples from TCGA and GEO databases. Before combining these two expression profiles, PCA was conducted; the result showed that these data form two databases could be merged properly (**Supplementary Figure S1**). Then, the optimal number of clusters was identified as 2 based on the immune cell infiltration (ICI) level (**Supplementary Figures 2A–C**). A total of 527 patients were divided into two subtypes with ICI clusters A and B (**Figure 2A**), and a significant survival difference was found between these two ICI clusters (log-rank test, p = 0.041, **Figure 2B**).

To further investigate the association between immanent biological features and diverse clinical phonotypes, we compared the immune cell composition of the TME between the two subtypes. ICI cluster A was characterized by poorer clinical outcome, which showed a higher level of naive B cells, memory B cells, CD4 memory resting T cells, activated natural killer (NK) cells, monocytes, M0 macrophages, M2 macrophages, resting dendritic cells, activated dendritic cells, activated mast cells, and neutrophils infiltration. ICI cluster B showed a more favorable prognosis performance and exhibited a greater fraction of plasma cells, CD8 T cells, activated CD4 memory T cells, follicular helper T cells, regulatory T cells (Tregs), gamma delta T cells, M1 macrophages, and resting mast cells (**Figure 2C**). It was visualized in the correlation coefficient heatmap to demonstrate the interaction of immune cell infiltration in TME (**Figure 2D**). In addition, the level of four prominent immune checkpoints, namely, cytotoxic T lymphocyte antigen-4 (CTLA-4), programmed cell death protein 1 (PD-1), programmed death ligand-1 (PD-L1), and programmed death ligand-2 (PD-L2), were analyzed in ICI clusters. The results featured that ICI cluster B manifested higher expression of CTLA4, PD1, PD-L1, and PD-L2 than ICI cluster A (**Figures 2E–H**). We found a higher performance in ICI cluster B using ESTIMATE score and immune scores, which can capture tumor purity and the infiltration of immune cells in tumor tissue (**Supplementary Figures S3A, C**). However, there was no obvious difference between these two clusters under stromal score (**Supplementary Figure S3B**).

### Identified Differentially Expressed Immune Gene Subtype

**Figure 3:**
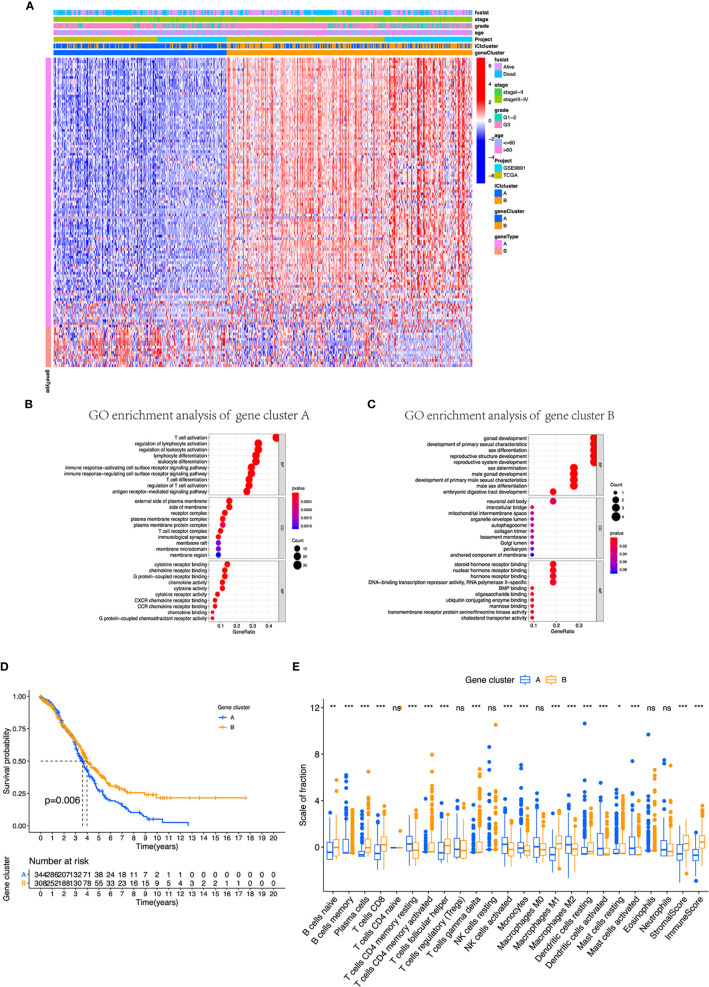
Identification of immunogenic gene subtypes in the TME of OC. **(A)** Unsupervised clustering of common DEGs derived from two ICI cluster groups helped divide patients into two subgroups: gene clusters A and B. **(B, C)** Gene Ontology (GO) enrichment analysis of the gene clusters A and B. **(D)** OS of all patients was compared between two gene clusters applying Kaplan–Meier curves. **(E)** Cellular fraction of the tumor-infiltrating immune cell types in gene clusters A and B, including stromal score and immune score. *p < 0.05; **p < 0.01; ***p < 0.001; ns, not significant.

We conducted differential expression analyses to distinguish the transcriptome variations between the two subtypes to comprehend biological features of different immunophenotypes. A total of 117 differentially expressed genes (DEGs) were obtained first, and unsupervised clustering categorized the cohort (TCGA and GSE9891) into two genomic clusters designated by gene cluster A and gene cluster B (**Figure 3A**, **Supplementary Figures S4A–C**). Furthermore, 102 signature genes that positively associated with the gene cluster were named as the ICI signature A and the remaining 15 as ICI signature B. We performed Boruta algorithm in the ICI clusters A and B to reduce the noise or redundant genes. GO enrichment analyses showed that in gene cluster A, T-cell activation, regulation of lymphocyte, leukocyte activation, and leukocyte differentiation were considered statistically significant in biological processes; the external side of plasma membrane, side of membrane, and receptor complex were found as important members of cellular components (**Figures 3B, C**). Cytokine receptor binding, G-protein-coupled receptor binding, and chemokine receptor binding were characterized as prominent ones in molecular function (**Figures 3B, C**). Meanwhile, gene cluster B was featured by enrichment in gonad development, development of primary sexual characteristics, neuronal cell body, steroid hormone receptor binding, and nuclear hormone receptor binding.

**Figure 4:**
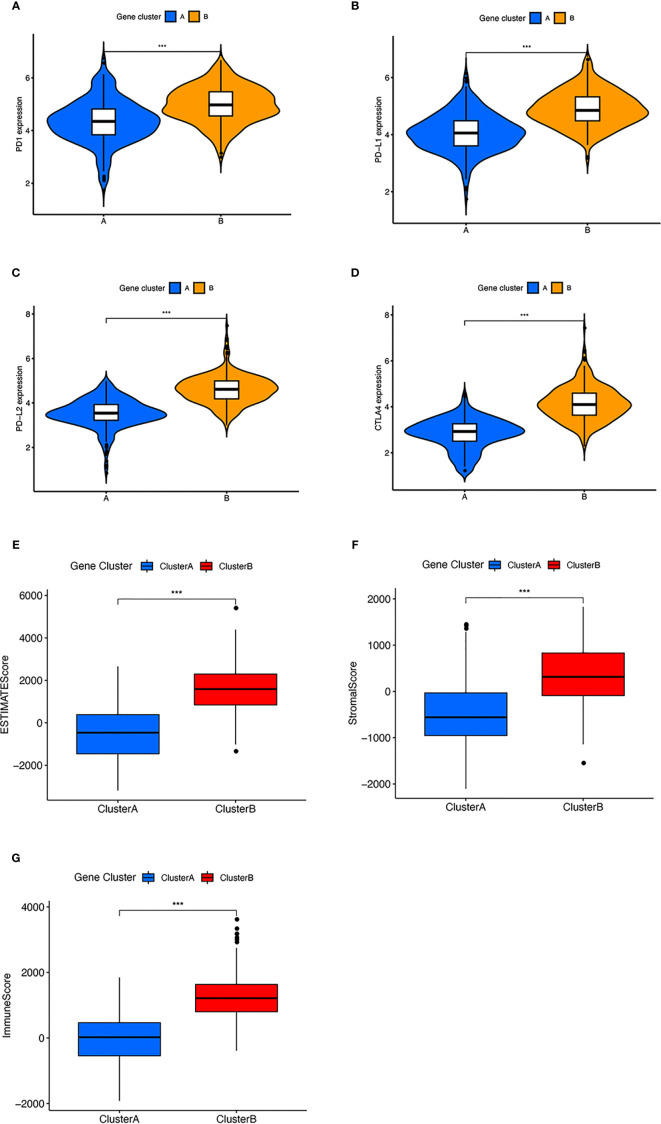
The expression of immune checkpoint and immune related score in two clusters. **(A–D)** Difference in **(A)** PD-1, **(B)** PD-L1, **(C)** PD-L2, and **(D)** CTLA4 expression between two gene clusters. **(E–G)** Difference in ESIMATE score, stromal score, and immune score between two gene clusters. ***p < 0.001.

Moreover, we integrated survival information with prognostic significance of the ICI gene clusters by exploiting the Kaplan–Meier plotter. We found that gene cluster B showed a longer overall survival than gene cluster A (log-rank test, p = 0.006, **Figure 3D**). In addition, gene cluster B possessed significantly elevated plasma cells, CD8 T cells, and M1 macrophages infiltration, while showed decreased resting memory CD4 T cells, monocytes, M2 macrophages, and activated DC cells (**Figure 3E**). We also compared the expression level of CTLA4, PD1, PD-L1, and PD-L2, which all displayed a greater level in gene cluster B (**Figures 4A–D**). These results were also consistent to what we found using ESTIMATE score, immune score, and stromal score (**Figures 4E–G**).

### Development and Evaluation of the ICI Score

**Figure 5:**
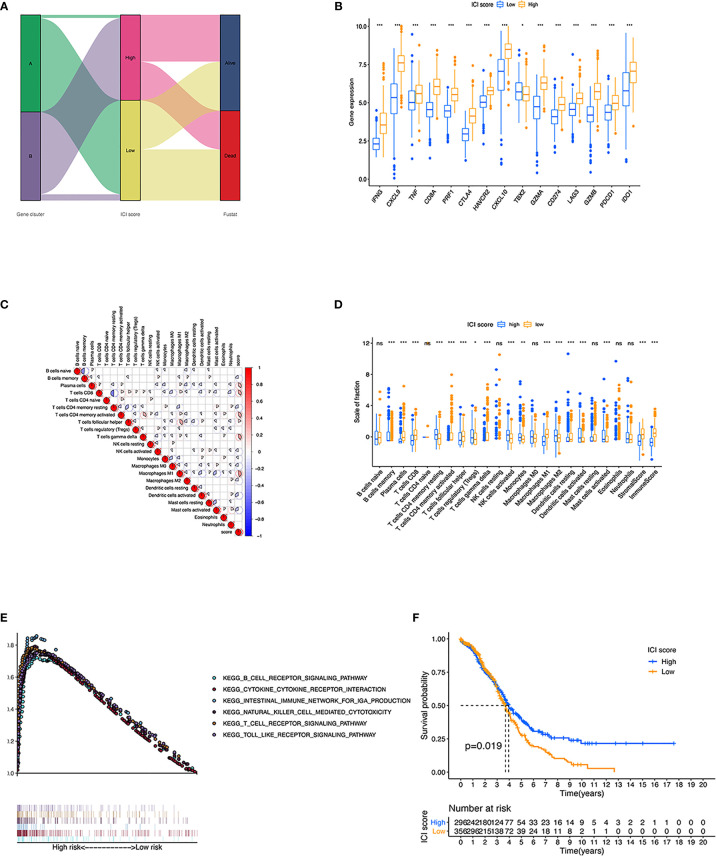
Construction of ICI scores. **(A)** Alluvial diagram of ICI gene cluster distribution in groups regarding to different gene clusters, ICI scores, and survival status. **(B)** Immune-activity-related signatures and immune-checkpoint-relevant signatures in low and high ICI subgroups. **(C)** Interaction of immune cell infiltration and ICI score in two gene clusters. **(D)** Cellular fraction of the tumor-infiltrating immune cell types in high and low ICI, including Stromal Score and Immune Score. **(E)** Gene set enrichment plots showed the rich KEGG pathways in the high ICI subgroup. **(F)** Kaplan-Meier curves for high and low ICI groups in two OC cohort. *p < 0.05; **p < 0.01; ***p < 0.001; ns, not significant.

To assess the comprehensive variables of ICI landscape in OC patients, we used PCA to calculate the ICI scores A and B from ICI signature genes A and B, respectively. ICI scores A and B were defined as the sum of individual relevant scores for each patient in the cohort. Eventually, construction of ICI score was obtained using the prognostic signature score. In this way, patients in the two datasets were classified into two subgroups, namely, the high ICI and low ICI (**Figures 5A, B**). To evaluate the immune activity of each group, we selected IFNG, CXCL9, TNF, CD8A, PRF1, CTLA4, HAVCR2, CXCL10, TBX2, and GZMZ as immune-activity-related signatures and CD274, LAG3, GZMB, PDCD1, and IDO1 as immune-checkpoint-relevant signatures. We observed a harbored significant overexpression in the high ICI group among all these related genes except TBX2. A correlation coefficient heatmap was drawn to demonstrate the interaction of immune cell infiltration in ICI scores (**Figure 5C**). The Cellular fraction of the tumor-infiltrating immune cell types, stromal score and immune score between two ICI groups were also compared (**Figure 5D**). The GSEA revealed that the high ICI group significantly enriched genes including B-cell receptor, cytokine receptor, intestinal immune network for IgA production, NK-cell-mediated cytotoxicity, T-cell receptor, and Toll-like receptor pathway (**Figure 5E**).

Then, we evaluated the prognostic value of the ICI scores, and patients with high ICI had a better clinical OS rate than those with low ICI (*p* = 0.019, **Figure 5F**). We also compared the prognosis of different subgroups based on the clinical information that patients in high ICI group had a greater chance to survival in subgroups of <60 years old, G3 grade, stage III–IV tumor, and whether they received chemotherapy or not. (**Supplementary Figure S5**). To expand the prognostic power of the ICI score and other clinical characteristics, we constructed a nomogram. Each parameter was assigned with a score, and their total score was calculated. With the help of the total score, 3-, 4-, and 5-year survival were predicted (**Supplementary Figure S6**).

Subsequently, we investigated the relationship between ICI score and the infiltration of immunocyte. The ICI score showed positive correlation with the infiltration of plasma cells, activated CD4 memory T cells, M1 macrophages, CD8 T cells, gamma delta T cells, resting mast cells, and follicular helper T cells (**Supplementary Figure S7**). However, a negative correlation was observed in activated NK cells, activated dendritic cells, monocytes, memory B cells, M0 and M2 macrophages, and activated mast cells (**Supplementary Figure S7**).

### The Association Between the ICI Score and Somatic Variants

**Figure 6:**
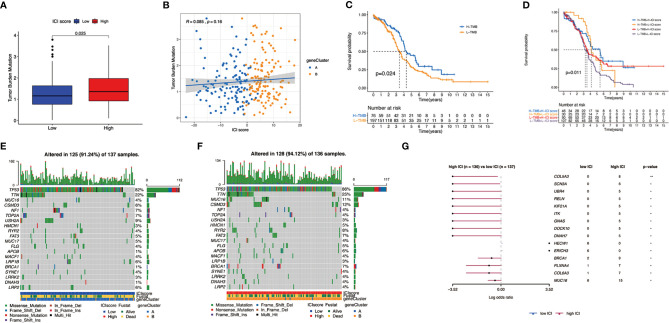
The association between the ICI scores and somatic variants. **(A)** Tumor mutation burden (TMB) difference in the high and low ICI subgroups. **(B)** Scatterplots describing the positive correlation between ICI scores and TMB in the OC cohort. **(C)** Kaplan–Meier curves for high and low TMB groups of the OC cohort. **(D)** Kaplan–Meier curves for patients in the OC cohort stratified by both TMB and ICI scores. **(E)** The single nucleotide variants (SNVs) were constructed using high ICI on the left (blue) and **(F)** low ICI on the right (red). Individual patients are represented in each column. **(G)** Differentially mutated genes between low ICI and high ICI displayed as a forest plot.

There have been some studies that implied that tumor mutation burden (TMB) could be a potential marker for patients’ response to immunotherapy (35–37). Patients in the high ICI group had significantly higher TMB than those in the low ICI group (*p* = 0.025) (**Figure 6A**). Moreover, ICI score had a positive relationship with TMB although with no obvious statistical significance (R = 0.085, *p* = 0.16, **Figure 6B**). Comparing the survival probability of patients who classified into high and low TMB groups, patients with high TMB were more likely to have a significantly ideal overall survival rate (log-rank test, p = 0.024, **Figure 6C**). Subsequently, we performed a stratified survival analysis that combined TMB score with ICI score, which indicated that high and low TMB subgroups classified according to ICI score showed a significant survival difference (log-rank test, *p* = 0.011, **Figure 6D**).

Apart from that, we mapped the landscape of somatic changes in ovarian cancer between the low and high ICI groups. Mutation data were analyzed using the maftools packages in R. We chose top 20 genes that were most likely to be mutated in high and low ICI, and the type of mutation like missense mutation, frame shift deletion, and nonsense mutation were also included (**Figures 6E, F**). Comparison of low and high ICI revealed 15 genes to be differentially mutated (**Figure 6G**).

### The Application of ICI Score in Predicting Immunotherapy

**Figure 7:**
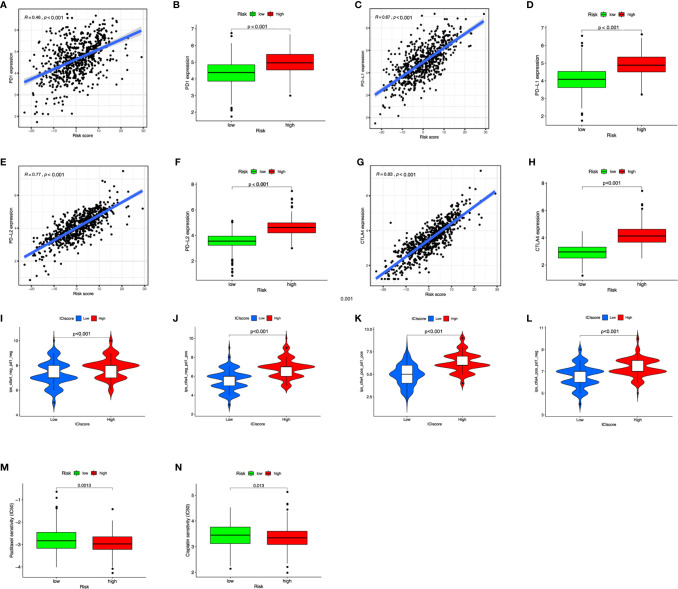
The benefits of ICI score in predicting immunotherapy. **(A–H)** Scatterplot and boxplot of PD1 **(A**, **B)**, PD-L1 **(C**, **D)**, PD-L2 **(E**, **F)**, and CTLA4 **(G**, **H)** expression in risk scores. **(I–L)** IPS values of patients categorized according to ICI score of four subtypes [IPS-CTLA4-neg-PD1-neg **(I)**, IPS-CTLA4-neg-PD1-pos **(J)**, IPS-CTLA4-pos-PD1-pos **(K)**, IPS-CTLA4-pos-PD1-neg **(L)**]. **(M**, **N)** The IC50 of cisplatin and paclitaxel in low and high ICI groups.

**Figure 8:**
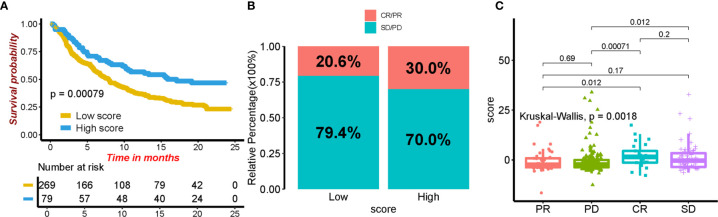
The role of ICI score in predicting the response to immunotherapy. **(A)** Kaplan–Meier curves for patients in different ICI score groups. **(B)** The rate of response to immunotherapy in high and low ICI groups. **(C)** The distribution of ICI score in different tumor status.

In our study, we assigned patients in the cohort to different subgroups based on the median ICI scores. In addition, the association of immune checkpoints and ICI score was provided. Not surprisingly, patients in the high ICI group showed a higher expression in CTLA4, PD1, PD-L1, and PD-L2 and were positively correlated with ICI score (**Figures 7A–H**). We categorized patients into four types (i.e., IPS-CTLA4-neg-PD1-neg, IPS-CTLA4-neg-PD1-pos, IPS-CTLA4-pos-PD1-neg, and IPS-CTLA4-pos-PD1-pos) according to their ICI score (**Figures 7I–L**). ESTIMATE score, immune score, and stromal score were all higher in the high ICI group (**Supplementary Figure S8**). It is obvious that patients with high ICI expressed greater IPS values. In order to investigate whether ICI score can predict the response to immunotherapy. An immunotherapy cohort (IMvigor210) was included. Patients with high ICI score showed better over survival (**Figure 8A**), and patients in the high ICI groups exhibited higher objective response rate than patients in the low ICI group (**Figure 8B**). Besides, we also compared the distribution of ICI score in different disease status. The ICI score in CR was significantly higher than that in PR, SD, and PD (**Figure 8C**), which confirmed that ICI score can predict the response to immunotherapy. Two first-line chemotherapy drugs (cisplatin and paclitaxel) for ovarian cancer also showed a higher sensitivity in high ICI subgroup (**Figures 7M, N**).

## Discussion

Early studies on immunotherapy in OC indicated its potential ability in acting as a compensating role apart from first-line treatment, including ICIs, cancer vaccines, and adoptive T-cell therapy (38, 39). However, considering that only 10–20% of patients respond to immunotherapy (anti-PD1 and anti-CTLA-4 treatments) in a recent clinical trial, no drugs were officially approved in practice until now (40, 41). OC was considered as an immunogenic tumor, and several non-spontaneous antitumor immune responses were detected in tumors. Such low response rate to this tumor was confusing and aroused great interest of scientific research. For patients suffering from OC, it is important to identify biomarkers that can predict response performance to immunotherapy. Therefore, we established an ICI score here to quantify the tumor immune environment in OC for predicting response to immunotherapy.

Many studies have defined OC as a potentially immunoreactive tumor (42–44). First, we divided patients into two immune subgroups (ICI clusters A and B) based on the immune cell infiltration level. Consistent with previous research, our study also demonstrated that CD4 memory resting T cells, activated NK cells, monocytes, M0 and M2 macrophages, dendritic cells, activated mast cells, and neutrophils were increased in ICI cluster A, which positively correlated with clinical outcomes (45).

We further obtained the different expression immune-related genes between ICI clusters A and B. Using unsupervised clustering of DEGs, we categorized patients into gene clusters A and B. In these two gene clusters, we observed that ICI gene cluster B had a higher immune score, ESTIMATE score, immune score, and inflammatory cells, which suggested that it is an immune-hot phenotype. Contrary to this, our results showed that gene cluster A presented relatively lower immune score, stromal score, and ESTIMATE score, which indicated that it is an immune-cold tumor. Based on the above analysis, it is easy to see that gene cluster B was associated with a higher density of inflammatory cells infiltration, like plasma cells, CD8^+^ T cells, and M1 macrophages. Immune hot tumors are associated with characteristics such as the expression of PD-L1 on tumor-associated immune cells, potential genomic instability, and the presence of a pre-existing antitumor immune responses (42–44). Apart from that, the relationship between patients’ OS and TME was clearly analyzed. Our results, in accordance with previous studies, showed that ICI gene cluster B, an immune-hot tumor, presented a better prognosis than gene cluster A, which is immunologically ignorant (scarcely expressing PD-L1). In order to make this classification system more suitable for clinical use, we introduced ICI signatures A and B, then constructed the ICI scoring system. With the help of the ICI scoring system, we can easily predict the overall survival of OC patients. Patients with high ICI score had better prognosis than those with low ICI score. GSEA and KEGG analyses were conducted to identify the different biological pathways; we observed enrichment in B-cell receptor, cytokine receptor, intestinal immune network for IgA production, NK-cell-mediated cytotoxicity, T-cell receptor, and Toll-like receptor signaling pathway in the high ICI group. Furthermore, the association between the tumor mutational burden and response to immunotherapy was identified in previous studies (46, 47). High TMB has positive correlation with sensitivity to immunotherapy (47). In our study, it was validated that the ICI score was positively correlated with TMB. Patients in the high ICI group showed higher TMB. The expression of immune-checkpoint molecules and IPS were the commonly used markers for predicting the response to immunotherapy (31). In this research, we found that patients in the high ICI groups exhibited higher expression of CTLA4, PD1, PD-L1, PD-L2, and greater IPS values. Based on the results of TMB, the expression of immune-checkpoint molecules, and IPS, patients in the high ICI group may benefit from immunotherapy. The ICI scoring system can predict the response to immunotherapy. At the same time, we also evaluated the IC50 of two first-line chemotherapy drugs applied frequently in ovarian cancer treatment; the results revealed that low ICI patients may not benefit from chemotherapy regimen based on these two agents. As a result, other chemotherapy drugs should be prepared.

In fact, there were several studies focused on predicting the prognosis of OC patients (48–52). In 2019, Shen et al. developed a prognostic signature based on 129 genes for OC and claimed its ability in predicting the prognosis of OC patients (48). However, this research included many genes that limited its clinical application. In 2020, Bao et al. established a similar signature based on only eight genes; researchers also verified its power in reflecting the prognosis information of OC patients (49). In the same year, only four prognosis-related genes were utilized by An et al. for building a signature (50). It was reported that the signature can reflect the survival information and the response to immunotherapy. Different from the existing research mainly focused on the expression of some specific genes. Our research stratifies patients into different groups by CIBERSORT algorithm. Then, the ICI score was established based on PCA analysis of the DEGs between the clusters. This process can reduce the infidelity of CIBERSORT results (53). This score system is shown to be more stable and may not be influenced by a single or several gene expression (54–56). Besides, the established ICI scoring system can also provide valuable information on designing of the optional therapeutic regimen.

Nevertheless, this study was mainly based on the data from online database. The fundamental research was essential for explaining the internal mechanism of this scoring system. Besides, clinical studies are still needed to validate the predictive value of ICI scoring system.

## Conclusion

In summary, we analyzed the landscape of ICI score in OC patients, painting a novel picture of regulation of immune response and immunotherapy, and confirmed its association with clinical outcome. We established a satisfying ICI score for predicting the prognosis of OC patients and providing the potential response to immunotherapy. With the help of ICI score, the clinicians can select suitable therapeutic regimens for patients.

## Data Availability Statement

The datasets presented in this study can be found in online repositories. The names of the repository/repositories and accession number(s) can be found in the article/**Supplementary Material**.

## Ethics Statement

Written informed consent was obtained from the individual(s) for the publication of any potentially identifiable images or data included in this article.

## Author Contributions

JB conceived the study and participated in the study design, performance, and manuscript writing. JL and YW conducted the bioinformatics analysis. SY and JW revised the manuscript. All authors contributed to the article and approved the submitted version.

## Conflict of Interest

The authors declare that the research was conducted in the absence of any commercial or financial relationships that could be construed as a potential conflict of interest.

## Publisher’s Note

All claims expressed in this article are solely those of the authors and do not necessarily represent those of their affiliated organizations, or those of the publisher, the editors and the reviewers. Any product that may be evaluated in this article, or claim that may be made by its manufacturer, is not guaranteed or endorsed by the publisher.

## Supplementary Material

Supplementary Figure S1 The principle-component analysis after conducting ComBat algorithm for two combined expression profiles.

Supplementary Figure S2 Consensus matrixes of all OC samples based on Infiltrating immune cells. **(A)** Cumulative distribution function curves for unsupervised clustering of OC based on Infiltrating immune cells, k = 2-9. **(B)** Relative change in area under the CDF curve for unsupervised clustering of OC, k = 2-9. **(C)** Heat map of the consensus matrix for the OC sample at k = 2.

Supplementary Figure S3 The distribution of three score in ICI cluster A and ICI cluster B. **(A)** ESTIMATE score. **(B)** Stromal score. **(C)** Immune score.

Supplementary Figure S4 consensus matrixes of all OC samples based on DEGs. **(A)** Cumulative distribution function curves for unsupervised clustering of OC based on DEGs, k = 2-9. **(B)** Relative change in area under the CDF curve for unsupervised clustering of OC, k = 2-9. **(C)** Heat map of the consensus matrix for the OC sample at k = 2.

Supplementary Figure S5 Prognostic value of ICI scores in OC cohorts. **(A**, **B)** Kaplan–Meier curves for patients with high and low ICI in patients <=60and >60 years old. **(C**, **D)** Kaplan–Meier curves for patients with high and low ICI in G1-2 patients and G3 patients. **(E**, **F)** Kaplan–Meier curves for patients with high and low ICI in patients with stage I-II and stage III-IV. **(G)** Kaplan–Meier curves for patients with high and low ICI in patients with wild BRCA1. (H) Kaplan–Meier curves for patients with high and low ICI in patients treated with chemotherapy.

Supplementary Figure S6 Construction and validation of a nomogram. **(A)** Nomogram to predict the probability of 3, 4 and 5-year OS of OC patients. **(B-D)** Calibration curves of the nomogram to predict the probability of OS at 3, 4 and 5 years.

Supplementary Figure S7 The correlation of immune cells and ICI scores. Scatterplots depicting the correlation between ICI scores and fraction of immune cells. **(A)** plasma cells, **(B)** CD4 memory T cells, **(C)** M1 macrophages, **(D)** CD8 T cells, **(E)** gamma delta T cells, **(F)** resting mast cells, **(G)** CD4 naïve T cells, **(H)** resting dendritic cells, **(I)** activated CD4 memory T cells, **(J)** activated NK cells, **(K)** activated dendritic cells, **(L)** monocytes, **(M)** follicular helper T cells, **(N)** memory B cells, **(O)** M0 macrophages, **(P)** activated mast cells, **(Q)** M2 macrophages in OC.

Supplementary Figure S8 The distribution of three score in high ICI and low ICI group. **(A)** ESTIMATE score. **(B)** Stromal score. **(C)** Immune score.
