# Supplementary figures and images for: Construction of an Immune Cell Infiltration Score to Evaluate the Prognosis and Therapeutic Efficacy of Ovarian Cancer Patients
Source: Front Immunol. 2021 Oct 20;12:751594. doi: 10.3389/fimmu.2021.751594 (PMC8564196; doi:10.3389/fimmu.2021.751594)

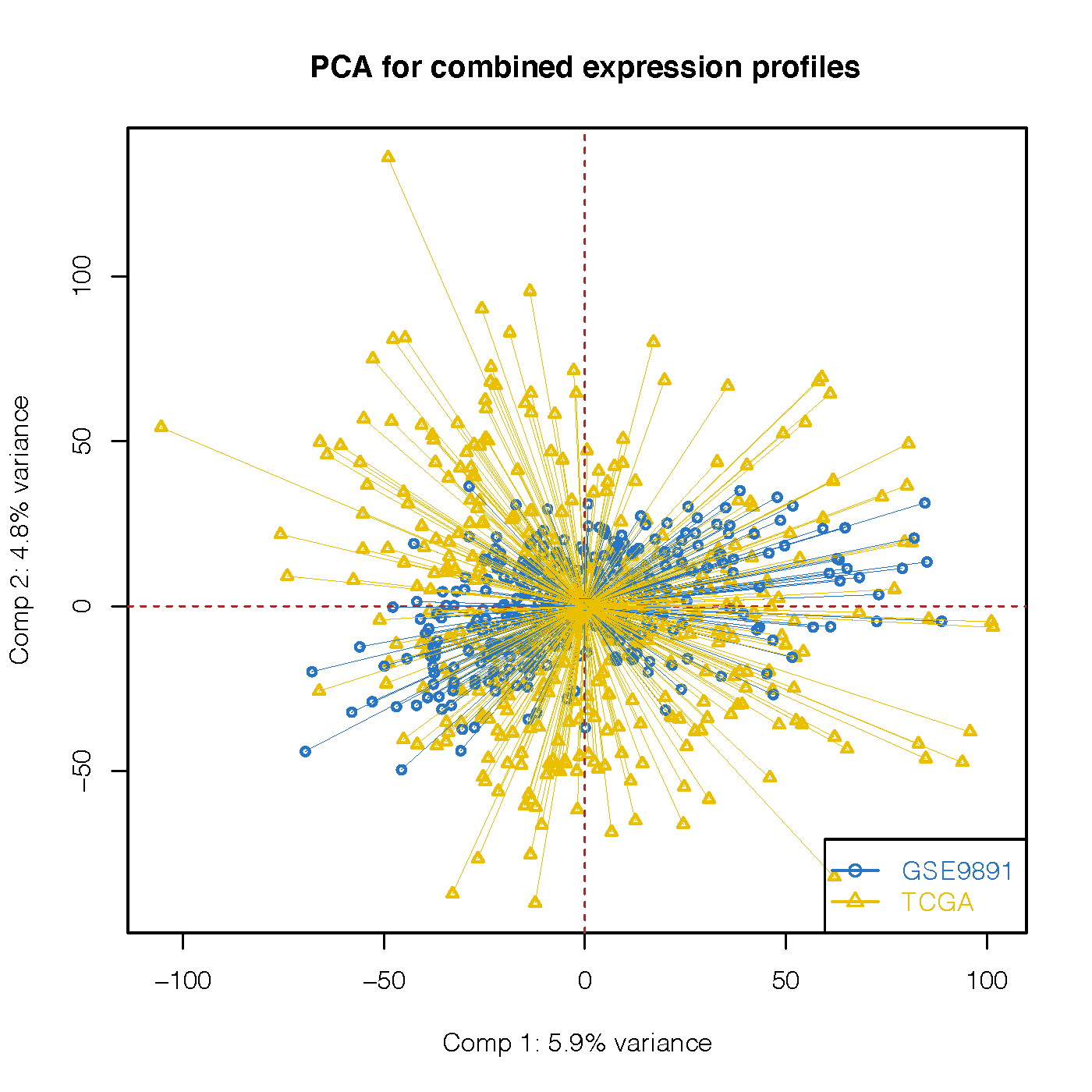

Supplement: Supplementary Figure S1 — The principle-component analysis after conducting ComBat algorithm for two combined expression profiles. [file Image_1.tiff]

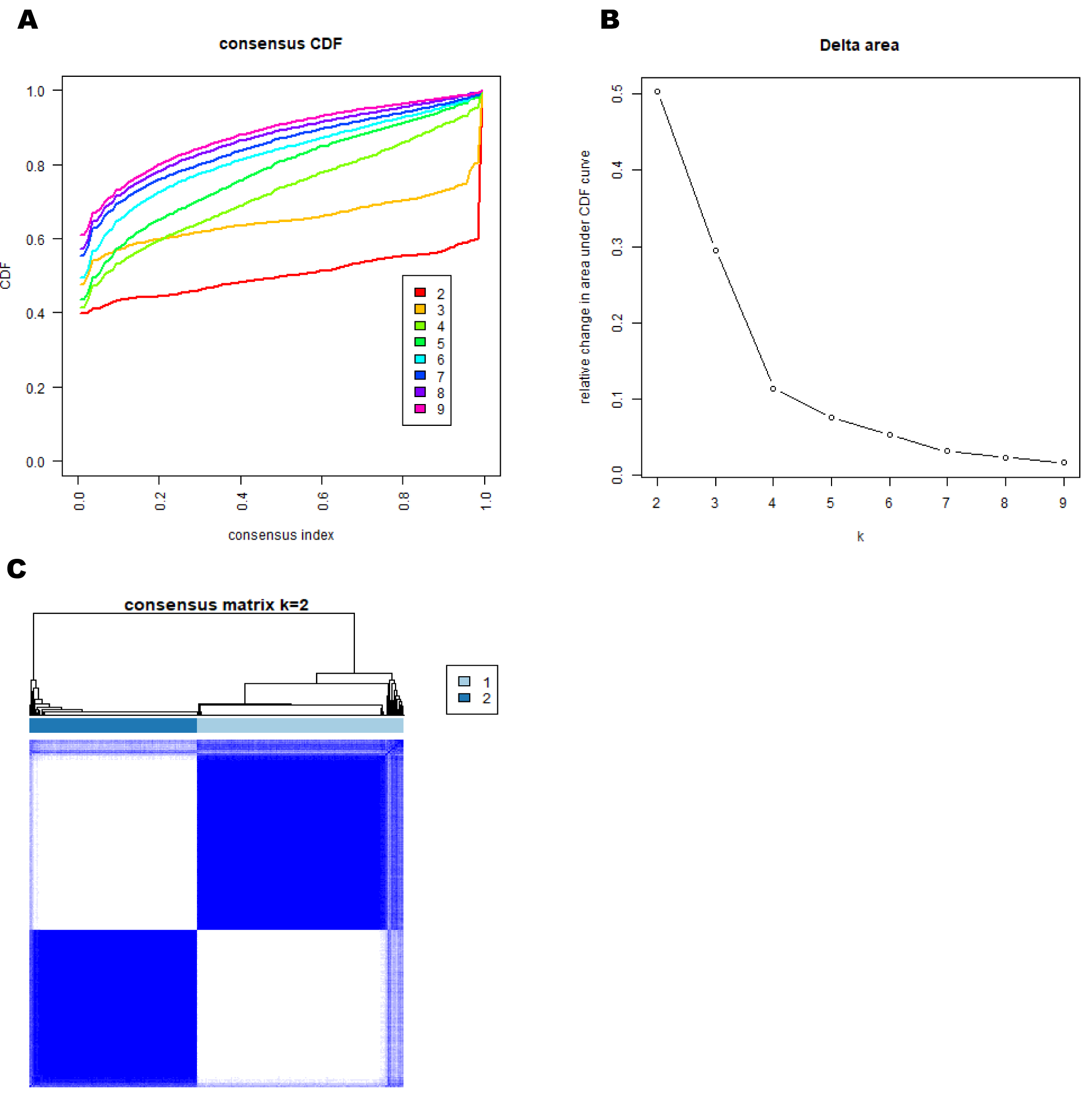

Supplement: Supplementary Figure S2 — Consensus matrixes of all OC samples based on Infiltrating immune cells. (A) Cumulative distribution function curves for unsupervised clustering of OC based on Infiltrating immune cells, k = 2-9. (B) Relative change in area under the CDF curve for unsupervised clustering of OC, k = 2-9. (C) Heat map of the consensus matrix for the OC sample at k = 2. [file Image_2.tif]

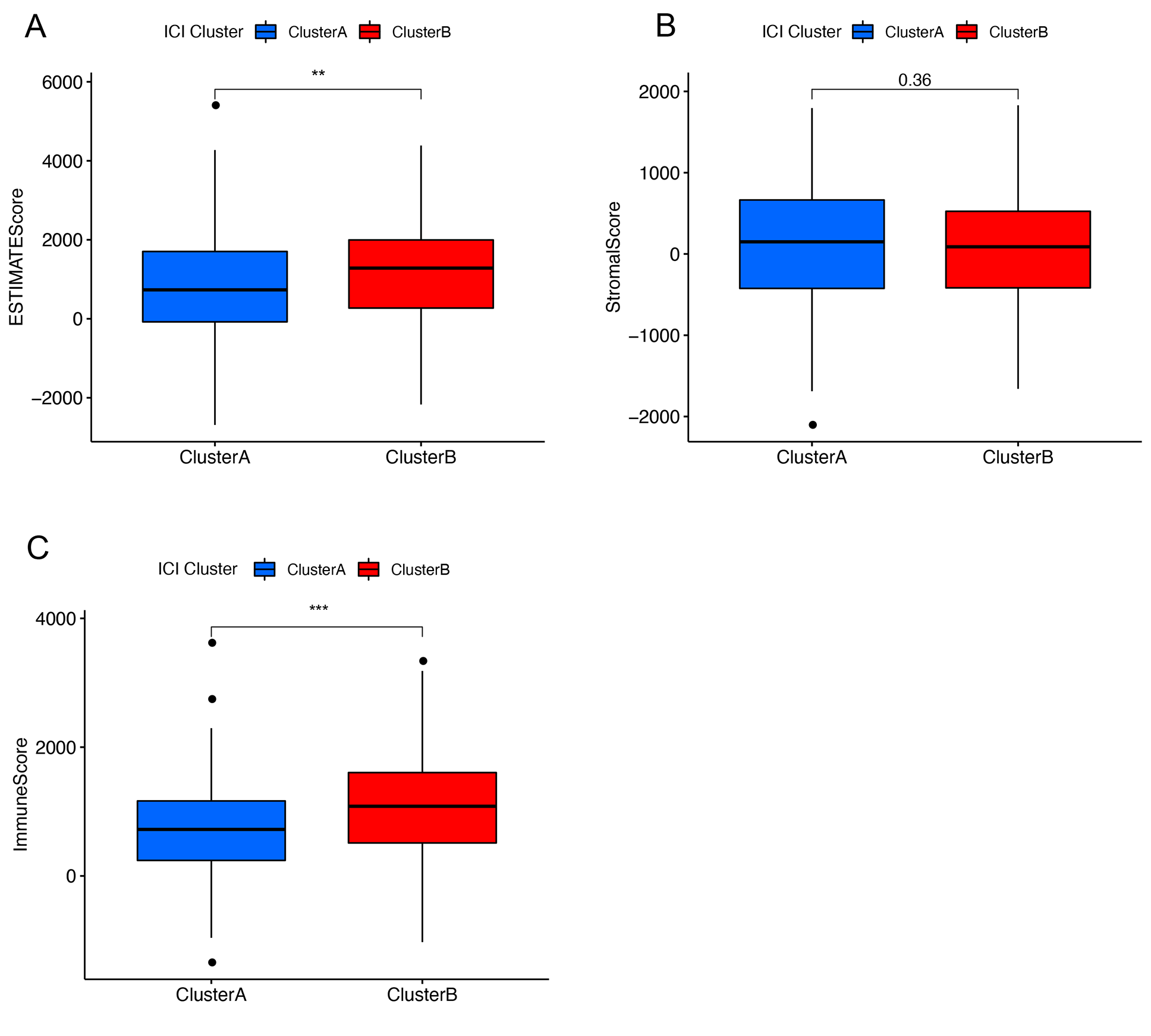

Supplement: Supplementary Figure S3 — The distribution of three score in ICI cluster A and ICI cluster B. (A) ESTIMATE score. (B) Stromal score. (C) Immune score. [file Image_3.tif]

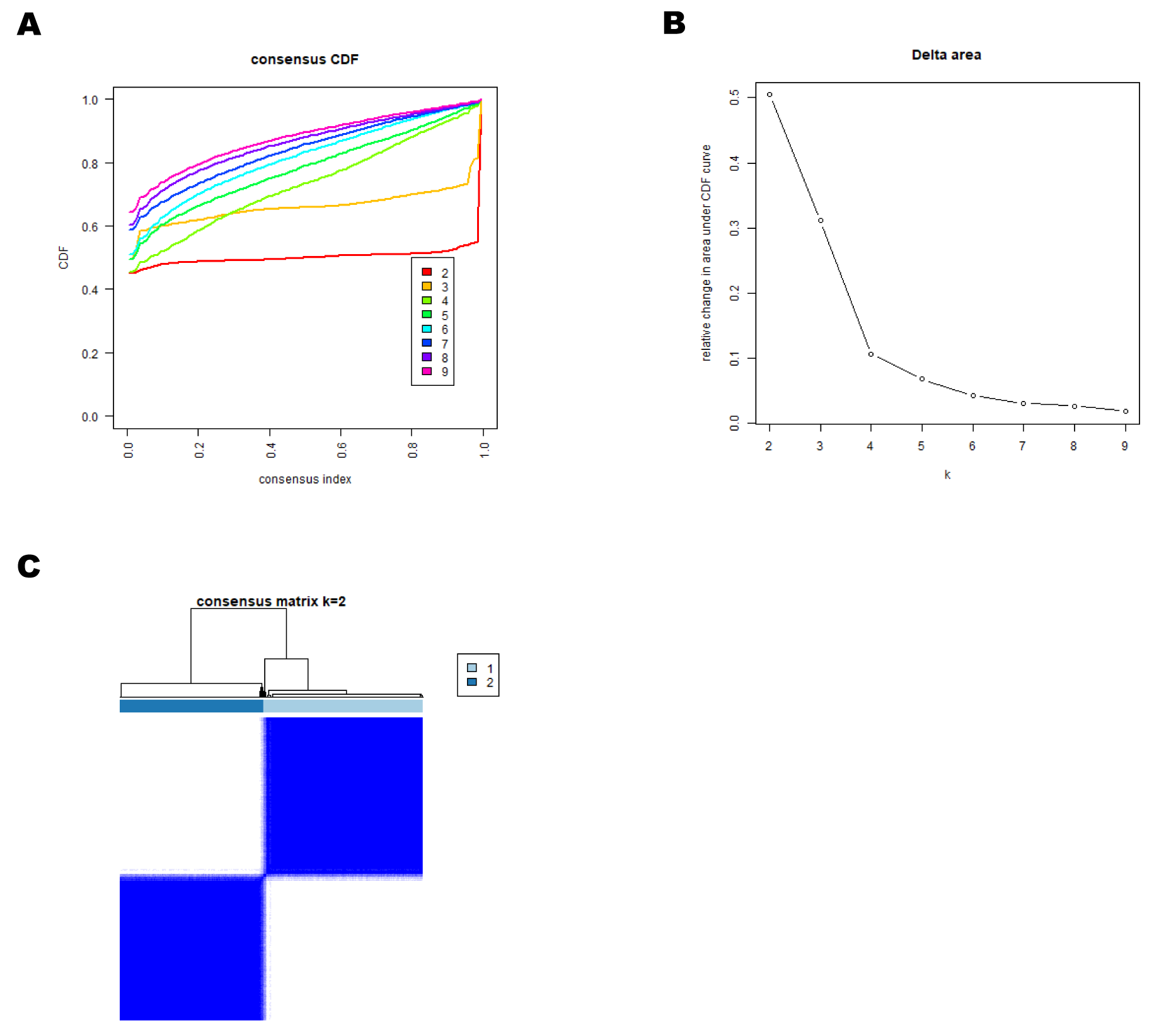

Supplement: Supplementary Figure S4 — consensus matrixes of all OC samples based on DEGs. (A) Cumulative distribution function curves for unsupervised clustering of OC based on DEGs, k = 2-9. (B) Relative change in area under the CDF curve for unsupervised clustering of OC, k = 2-9. (C) Heat map of the consensus matrix for the OC sample at k = 2. [file Image_4.tif]

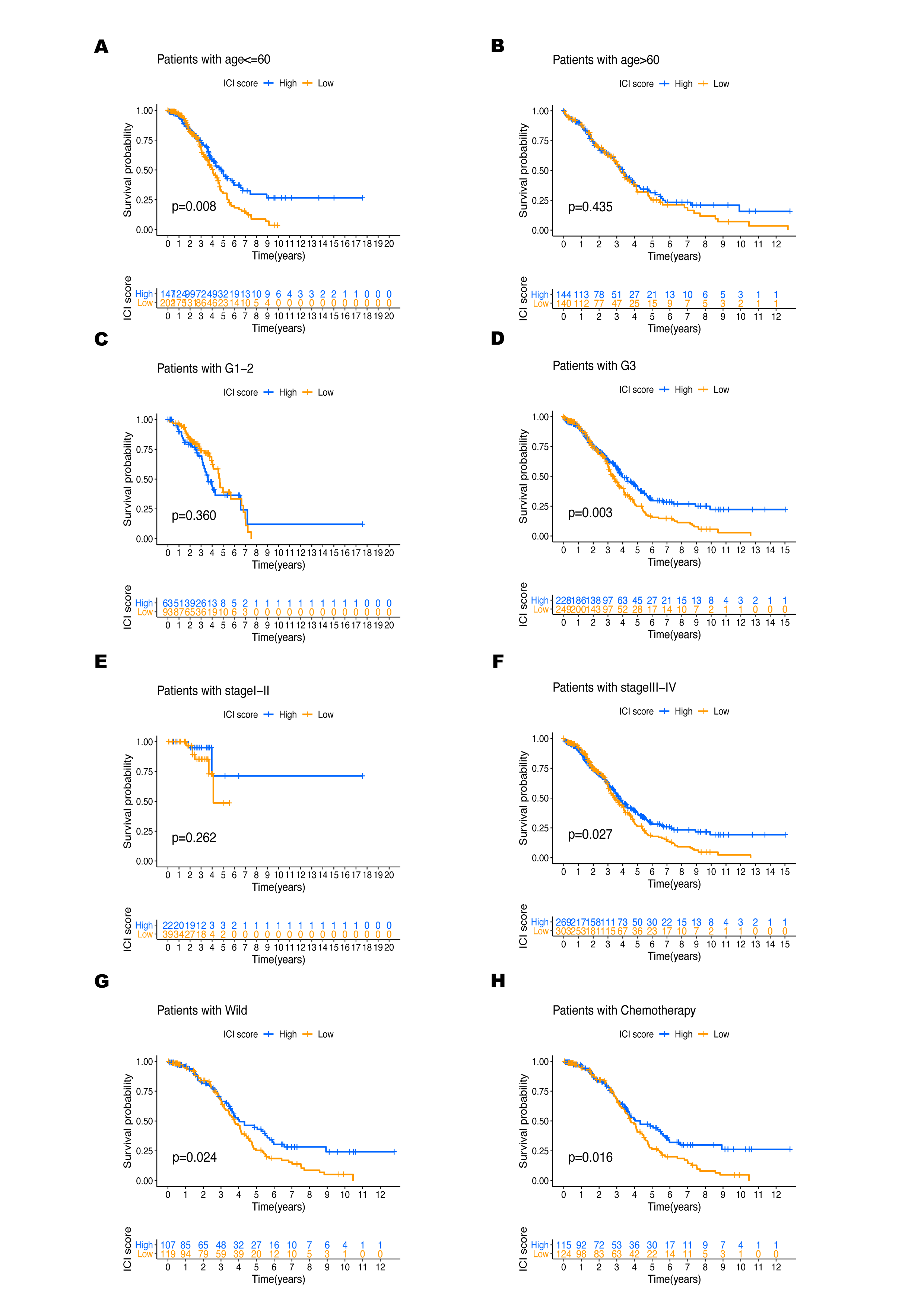

Supplement: Supplementary Figure S5 — Prognostic value of ICI scores in OC cohorts. (A, B) Kaplan–Meier curves for patients with high and low ICI in patients <=60and >60 years old. (C, D) Kaplan–Meier curves for patients with high and low ICI in G1-2 patients and G3 patients. (E, F) Kaplan–Meier curves for patients with high and low ICI in patients with stage I-II and stage III-IV. (G) Kaplan–Meier curves for patients with high and low ICI in patients with wild BRCA1. (H) Kaplan–Meier curves for patients with high and low ICI in patients treated with chemotherapy. [file Image_5.tif]

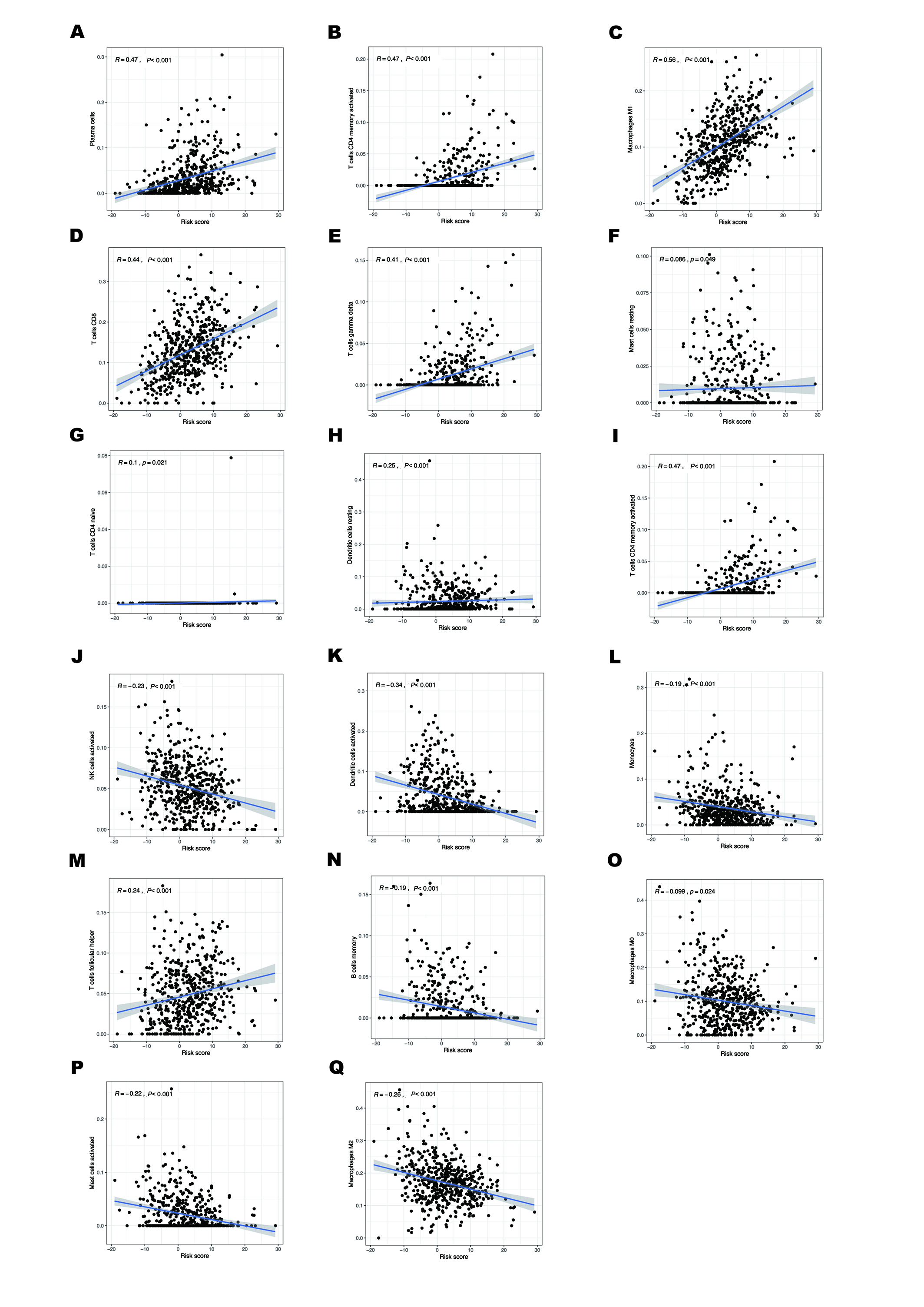

Supplement: Supplementary Figure S7 — The correlation of immune cells and ICI scores. Scatterplots depicting the correlation between ICI scores and fraction of immune cells. (A) plasma cells, (B) CD4 memory T cells, (C) M1 macrophages, (D) CD8 T cells, (E) gamma delta T cells, (F) resting mast cells, (G) CD4 naïve T cells, (H) resting dendritic cells, (I) activated CD4 memory T cells, (J) activated NK cells, (K) activated dendritic cells, (L) monocytes, (M) follicular helper T cells, (N) memory B cells, (O) M0 macrophages, (P) activated mast cells, (Q) M2 macrophages in OC. [file Image_7.tif]

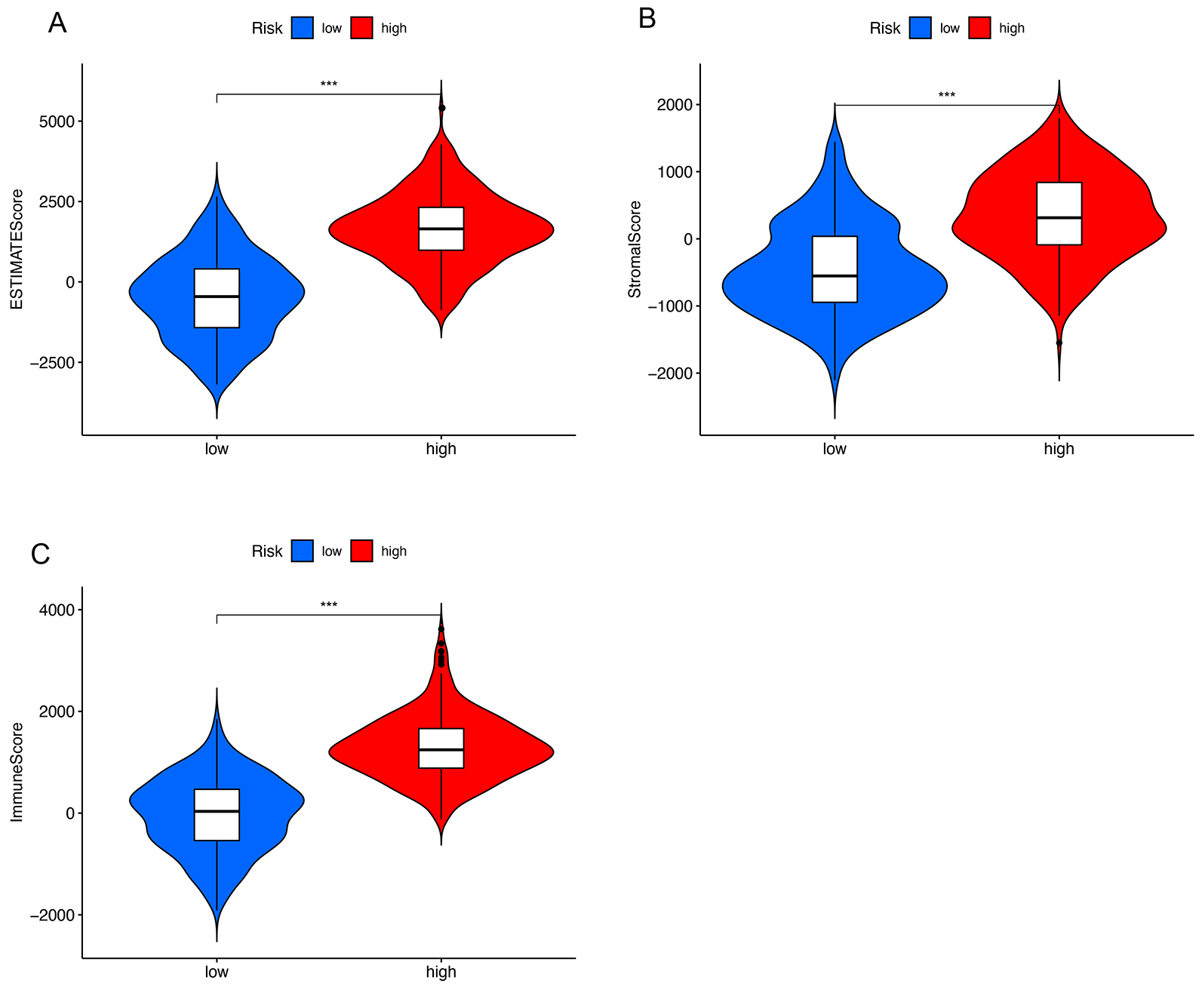

Supplement: Supplementary Figure S8 — The distribution of three score in high ICI and low ICI group. (A) ESTIMATE score. (B) Stromal score. (C) Immune score. [file Image_8.tif]
